# Efficacy and Cost over 12 Hospitalization Weeks of Postacute Care for Stroke

**DOI:** 10.3390/ijerph20021419

**Published:** 2023-01-12

**Authors:** Hsiang-Yun Chou, Ya-Wen Tsai, Shang-Chun Ma, Shang-Min Ma, Chia-Li Shih, Chieh-Ting Yeh

**Affiliations:** 1 Department of Rehabilitation, An Nan Hospital, China Medical University, Tainan 709204, Taiwan; 2 Institute of Physical Education, Health & Leisure Studies, National Cheng Kung University, Tainan 701401, Taiwan; 3 Department of Recreational Sport & Health Promotion, National Pingtung University of Science & Technology, Pingtung 912301, Taiwan; 4 Department of Nursing, An Nan Hospital, China Medical University, Tainan 709204, Taiwan

**Keywords:** postacute care, stroke, rehabilitation, functional outcome, walking ability, national health insurance cost

## Abstract

Few studies have investigated changes in functional outcomes and economic burden in patients in the postacute care cerebrovascular disease (PAC-CVD) program. We, for the first time, retrospectively investigated changes in functional performance and the national health insurance (NHI) cost over 12 PAC-CVD hospitalization weeks and evaluated the therapeutic effects of the PAC-CVD program on the NHI cost. Specifically, the functional outcomes and NHI cost of 263 stroke patients in the PAC-CVD program were analyzed. The repeated measures *t* test was used to compare functional performance over 0–3 weeks, and a one-way repeated measures multivariate analysis of variance was used to compare functional performance and NHI costs during weeks 0–6 and 0–9. The Wilcoxon signed-rank test was used to compare functional performance over weeks 9–12. Hierarchical multiple regression was used to estimate the effects of functional performance on NHI costs during weeks 3, 6, and 9. Over weeks 0–12, all functional performance measures demonstrated significant improvements. Changes in NHI costs varied depending on whether hospitalization was extended. At any time point, functional performance did not have a significant impact on NHI cost. Therefore, the PAC-CVD program may aid patients with stroke in sustainably regaining functional performance and effectively controlling economic burden.

## 1. Introduction

Annually, approximately 17 million stroke events occur worldwide; in other words, on average, a stroke event occurs every 2 s [1]. The advancement of medical technology and the promotion of public health policies have led to a decrease in stroke mortality; as such, the number of stroke survivors is expected to increase in the next 20 years, which may result in a considerable personal, social, and economic burden [2].

According to Taiwan national health insurance (NHI) data, 26,098 patients were hospitalized due to acute stroke in 2011, of which 16.2% had a prolonged (>30-day) length of stay (LOS), accounting for 56.4% of NHI claims for inpatient care. The NHI Administration (NHIA) launched a nationwide postacute care (PAC)-cerebrovascular disease (CVD) program in 2014; it provides three to five sessions of daily inpatient high-intensity rehabilitation facilities under per diem reimbursement to reduce medical care expenditure [3]. In addition, the PAC-CVD program emphasizes transition services: patients with stroke are required to be transferred to a community hospital (i.e., regional or district hospital) for the PAC-CVD program after receiving acute medical treatment at the medical center. Under the PAC-CVD program, LOS in a community hospital can be 3–6 weeks; it can be extended to up to 12 weeks by applying to the NHIA.

The PAC-CVD program has significantly improved the functional abilities, activities of daily living, and quality of life of patients with stroke in Taiwan [4,5,6,7]. Even during the COVID-19 pandemic, the quality and stability of the PAC-CVD program in Taiwan remained high, improving functional outcomes in patients with stroke [8]. The rates of nasogastric tube and urinary catheter removal at discharge were 66.7% and 90%, respectively [4]. Chu et al. recently investigated the effects of the PAC-CVD program in facilitating walking ability recovery and reported that the proportion of patients unable to complete the 5-m walking speed (5MWS) test decreased from 47.8% at admission to 8.6% at discharge [9]. Regaining walking ability is a major goal of poststroke rehabilitation because it is integral to improving the performance of the activities of daily living as well as increasing the likelihood of discharge, return to the household, and participation in community activities [10,11]. The PAC-CVD program uses the per diem reimbursement system, which significantly reduces hospitalization costs compared with the conventional fee-for-service reimbursement system [12]. After discharge, the annual total direct medical cost in the PAC-CVD group was significantly lower than that in the conventional group [12,13]. Thus, the PAC-CVD program can significantly improve patient functional performance and walking ability, thereby reducing medical costs. However, the impact of functional recovery and regaining walking ability during PAC-CVD hospitalization on NHI costs remains unclear. Therefore, in this study, we first explored the changes in functional ability, walking ability, and NHI cost for different hospitalization durations and then investigated the effects of functional recovery and regaining walking ability on NHI costs.

## 2. Materials and Methods

### 2.1. PAC-CVD Program

Patients are included in the PAC-CVD program if they (1) had an acute stroke ≤1 month and are in a stable medical status; (2) have moderate to moderately severe functional impairment with a modified Rankin scale (mRS) score of 3–4; and (3) have active rehabilitation potential, sufficient physical ability, basic cognition, learning ability, and willingness.

Under the PAC-CVD program, patients are treated by a multidisciplinary PAC-CVD stroke team for integrated care; this includes doctors, nurses, physical therapists, occupational therapists, speech therapists, social workers, dietitians, and case managers. This team regularly assesses physical status—including motor function, activities of daily living, speech ability, swallowing ability, nutritional status, and quality of life—at the beginning of hospitalization, at discharge, and every 3 weeks during hospitalization.

The LOS is 3–6 weeks. If a patient needs to be hospitalized for >6 weeks, the multidisciplinary team’s conference records are sent to the NHIA, which decides whether and for how long the extension can be permitted. During hospitalization, the PAC-CVD program uses per diem reimbursement of TWD 3645, which includes fees for high-intensity rehabilitation (3–5 sessions per day), inpatient consultation, ward, nursing, and medical examination.

### 2.2. Study Design and Patients

This was a retrospective observational study, where we collected the data of patients with stroke who were included in the PAC-CVD program in a regional hospital in Southern Taiwan from January 2017 to June 2021. Specifically, we collected the following variables from patient medical records: patient demographics (age and sex); clinical attributes (stroke type, hypertension, coronary artery disease, diabetes mellitus, hyperlipidemia, and stroke history); common risk factors (smoking and drinking); functional performance measures [mRS score, Barthel index (BI), 5MWS, and 6-min walking distance (6MWD)] at prerehabilitation and inpatient rehabilitation weeks 3, 6, 9, and 12; PAC-CVD LOS; and NHI cost (in 2020, USD 1 = TWD 29.6).

The study protocol was approved by the Research Ethics Committee of Taiwan Municipal An Nan Hospital-China Medical University (TMANH110-REC007); the requirement for informed consent was waived because of the retrospective design of this study.

### 2.3. Functional Performance Measures

The mRS is a 0–6 point scale, widely used by clinical medical personnel to determine the general disability level of patients with stroke. The mRS scores of 0, 1, 2, 3, 4, 5, and 6 are defined as no symptoms, no significant disability, slight disability, moderate disability, moderately severe disability, severe disability, and death, respectively [14]. The higher the score is, the higher the degree of disability.

The BI includes 10 items to evaluate the performance of activities of daily living and self-mobility. The total score of the 10 items ranges from 0 to 100 (0–20 suggests total dependence, 21–60 severe dependence, 61–90 moderate dependence, and 91–99 slight dependence) [15,16]; the higher the score, the higher the independence of daily living.

The 5MWS test is used as a tool for evaluating the walking ability of patients with stroke. It measures the time taken to walk 5 m without assistance and calculates the walking speed; the higher the speed, the better the walking ability. Patients with stroke with a walking speed of <0.3 m/s may require inpatient rehabilitation intervention, whereas those with a walking speed of >0.6 m/s can be discharged from the hospital [17].

The 6MWD is an assessment of cardiorespiratory endurance and walking ability. It measures the distance completed by walking independently in 6 min without personal assistance. A 6MWD of ≥205 m is a predictor for the ability of a patient with a stroke to ambulate in the community [18].

### 2.4. Statistical Analysis

The patient sex, clinical attributes, and common risk factors were coded as categorical variables. In contrast, the patient age, PAC-CVD LOS, mRS score, BI, 5MWS, 6MWD, and NHI cost were coded as continuous variables and are presented as the means and standard deviations (*SD*s).

The repeated measures *t* test was used to compare functional performance prerehabilitation and at week 3. The one-way repeated measures multivariate analysis of variance (MANOVA) was used to compare functional performance and the NHI cost during weeks 0–6 and 0–9; the significance levels during weeks 0–6 and 0–9 were set at 0.0167 and 0.0125, respectively. The Wilcoxon signed-rank test was used to compare functional performance between weeks 9 and 12, with the data presented as the medians and interquartile ranges.

Hierarchical multiple regression was used to estimate the effects of functional performance during weeks 3, 6, and 9 on the NHI cost. Considering the difference in individual recovery abilities resulting in different functional performance prognoses, the patient demographics (age and sex), clinical attributes (stroke type, hypertension, coronary artery disease, diabetes mellitus, hyperlipidemia, and stroke history), and common risk factors (smoking and drinking) were set as the control variables. For each regression model, the variables were entered sequentially in three steps: (1) patient demographics (age, sex); (2) clinical attributes (stroke type, stroke history, hypertension, coronary artery disease, diabetes mellitus, and hyperlipidemia) and common risk factors (smoking and drinking); and (3) functional performance (mRS score, BI, 5MWS, and 6MWD). Normality was examined before conducting each hierarchical multiple regression; the absolute value of skewness should be below 2, and the absolute value of Kurtosis should be below 7 [19]. To confirm the multicollinearity problem of each regression analysis, we calculated the variance inflation factor (VIF). A VIF of >5 was considered to indicate the presence of a multicollinearity problem, which warrants resolution [20].

All the statistical analyses were conducted using SPSS (version 26.0; IBM Corp., Armonk, NY, USA). All the tests were two-sided, and a *p* of <0.05 was considered to indicate statistical significance.

## 3. Results

### 3.1. Patient Characteristics

**Figure 1:**
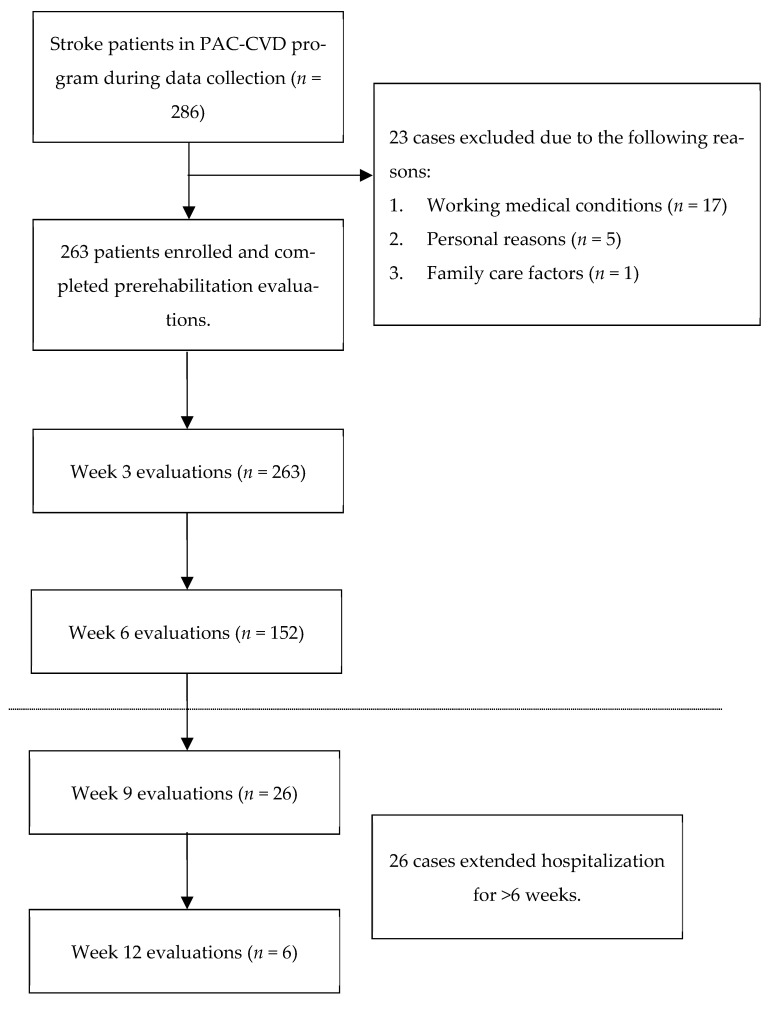
Flow chart of patient recruitment and data collection.

As shown in Figure 1, 286 patients were included in the PAC-CVD program at the regional hospital in Southern Taiwan during the data collection period. Of those patients, 17 dropped out due to working medical conditions, 5 requested withdrawal due to personal reasons, and 1 was suspended due to family care factors. Finally, 263 patients were enrolled in the present study and completed all the evaluations during hospitalization. We collected functional performance measures data of 263, 152, 26, and 6 patients during weeks 3, 6, 9, and 12, respectively. Of those patients, 26 had an extended LOS of >6 weeks.

As listed in Table 1, the mean (*SD*) age of the included patients was 63.74 (13.08) years. Most of the patients had an ischemic stroke, and >90% of the patients had a history of hypertension. The mean (*SD*) LOS was 32.56 (0.47) days, and most of the included patients had a LOS of 3–6 weeks. Under the per diem reimbursement system, the mean (*SD*) NHI cost was USD 3189.95 (USD 1501.07).

### 3.2. Functional Recovery and NHI Cost

#### 3.2.1. Patients with PAC-CVD LOS of at Least 3 Weeks

In total, 263 patients had a PAC-CVD LOS of at least 3 weeks. Compared with the baseline, all the functional performance measures demonstrated significant improvements at week 3. The mean (*SD*) NHI cost was USD 1895.31 (USD 251.47; Table 2).

#### 3.2.2. Patients with a PAC-CVD LOS of at Least 6 Weeks

In total, 152 patients had a PAC-CVD LOS of at least 6 weeks and completed baseline and week 3 and 6 evaluations. The one-way repeated measures MANOVA results demonstrated a significant main effect on functional performance measures and NHI cost at the evaluation time points (Wilk’s Lambda = 0.002, *F*_(10, 142)_ = 8695.86, *p* < 0.001, η_p_^2^ = 0.998; Table 3). A significant improvement occurred in all functional performance measures over weeks 0–6. The NHI cost was significantly higher during weeks 0–3 (mean = USD 2014.74, *SD* = USD 84.32) than during weeks 4–6 (mean = USD 1843.27, *SD* = USD 291.97; *p* < 0.001).

#### 3.2.3. Patients with a PAC-CVD LOS of at Least 9 Weeks

In total, 26 patients had a PAC-CVD LOS of at least 9 weeks and completed the baseline and week 3, 6, and 9 evaluations. The one-way repeated measures MANOVA results demonstrated a significant main effect on functional performance measures and the NHI cost at the evaluation time points (Wilk’s Lambda < 0.001, *F*_(15, 11)_ = 3862.59, *p* < 0.001, η_p_^2^ = 1.00; Table 4). All functional performance continued to improve significantly over 0–9 weeks. The NHI cost was significantly higher during weeks 4–6 (mean = USD 2060.84, *SD* = USD 44.57) than during weeks 7–9 (mean = USD 1928.40, *SD* = USD 178.61, *p* = 0.002) and 0–3 (mean = USD 1993.31, *SD* = USD 92.54, *p* = 0.001).

#### 3.2.4. Patients with a PAC-CVD LOS of 10–12 Weeks

Six patients had a PAC-CVD LOS of 10–12 weeks, and they completed all the evaluations. Their BI and 6MWD scores were significantly higher at week 12 than at week 9. Although the differences in the mRS and 5MWS scores were nonsignificant, the values tended toward improvement. The mean (*SD*) NHI cost during weeks 10–12 was USD 1695.53 (USD 264.65; Table 5).

#### 3.2.5. Impact of Functional Performance on NHI Cost

At week 3 of the PAC-CVD program, 16.6% of the variance in the cost of NHI was explained by the final regression model (*R*^2^ = 0.166, *F*_(14, 248)_ = 3.517, *p* < 0.001). The results of our hierarchical multiple regression analysis regarding the impact of functional performance on NHI cost demonstrated that age and sex had significant effects on NHI cost, each contributing 19.4% (β = −0.194) and 13.3% (β = 0.133) to the regression model, respectively. Thus, the results revealed that the higher the age, the lower the NHI cost at week 3. In terms of sex, the NHI cost was higher in male patients than in female patients (Table 6).

The hierarchical multiple regression analysis demonstrated no significant effect on the NHI cost at both PAC-CVD program weeks 6 and 9 [*F*_(14, 137)_ = 1.512 (*p* = 0.114) and *F*_(14, 11)_ = 1.669 (*p* = 0.199), respectively]. Notably, the predictors for functional performance (mRS, BI, 5MWS, and 6MWD) during weeks 3, 6, and 9 demonstrated no significant effect on the NHI cost.

## 4. Discussion

This was the first study to investigate the changes in functional performance, walking ability, and NHI cost of PAC-CVD patients over all 12 weeks of a hospital stay. Furthermore, we noted the impact of the therapeutic effect of the PAC-CVD program on the NHI cost for the first time. Our results demonstrated significant improvements in functional performance and walking ability at each evaluation time point. The NHI cost varied by the LOS. In patients with a PAC-CVD LOS of 0–6 weeks, the NHI cost was significantly higher during weeks 0–3 than during weeks 4–6. In patients with an extended PAC-CVD LOS (0–9 weeks), the NHI cost was the highest during weeks 4–6. At week 3, age and sex had significant explanatory power for the NHI cost. However, the functional performance measures did not have significant explanatory power for the NHI cost at any evaluation time point.

In Taiwan, the PAC-CVD program aims to aid patients in regaining functional abilities and reducing medical costs in patients with stroke who represent a potential window for recovery. Our findings were analogous to those of studies that investigated the PAC-CVD high-intensity rehabilitation of motor function [4,5,21,22] and observed the complete picture of the changes in functional performance and walking ability over 12 weeks of hospitalization. Most previous studies have focused on functional recovery at admission and discharge; however, none of them has analyzed changes in the functional performance continuum across weeks of hospitalization [9,21,22,23]. The current findings indicated that participating in the PAC-CVD program effectively improves the functional performance and walking ability of patients with stroke; they also demonstrated a trend of continuous improvement during 0–12 weeks of hospitalization. However, a lack of significant differences in the mRS and 5MWS scores between 9 and 12 weeks of hospitalization may be related to the small sample size included in this study. Moreover, mRS is used to assess the general level of functional impairment of daily living and the degree of walking assistance in patients with stroke [14,24]. In contrast, BI is used to evaluate independence in performing the activities of daily living, including feeding, bathing, grooming, dressing, bowel and bladder control, toilet use, transfer and mobility, and stair use; therefore, it can more realistically report changes in functional ability and self-mobility [16,25,26].

Walking disability is a major concern in patients with stroke because it is integral to performing the activities of daily living, which affects the quality of life [10,27]. In the current study, 5MWS demonstrated no significant improvement between 9 and 12 weeks. This finding might be related to the need to combine muscle strength, dynamic balance, weight bearing on the paretic side, and lower extremity motor coordination for gait speed [28,29,30]. Furthermore, regarding mental factors, the correlation between walking speed and gait confidence is generally moderate to strong [31,32]. In other words, patients in PAC require tailored rehabilitation strategies to facilitate walking recovery, including physical and psychological interventions. In the current study, 6MWD demonstrated a significant improvement between 9 and 12 weeks; this result might be associated with the high-intensity rehabilitation delivered through the PAC-CVD program. The patients in this program receive three to five rehabilitation sessions per day and, thus, they require more physical tolerance to adapt and demonstrate relatively high endurance and cardiorespiratory capacity during walking.

The PAC-CVD program is considered a more economical and favorable recovery strategy than traditional rehabilitation [12,33]. Chen et al. recently reported that within the first year after stroke rehabilitation, the NHI cost was higher in the non-PAC group (USD 3785 per person) than in the PAC group (USD 3480 per person) [13]. In the current study, the mean NHI cost during PAC-CVD hospitalization was USD 3189.95 (*SD* = USD 1501.07), and changes in the NHI cost varied depending on whether PAC-CVD hospitalization was extended. Of all the patients with stroke with at least 6 weeks of PAC-CVD hospitalization, the NHI cost was significantly higher during weeks 0–3 than during weeks 4–6. Under the per diem reimbursement system, the average LOS was lower during weeks 4–6 than during weeks 0–3. Thus, the patients with stroke were discharged before they stayed all of the 6 weeks. The decision to discharge may be related to several patient factors, including age, stroke severity, prestroke functional status, dementia, physical and cognitive function, mental status, comorbidities, social and family supports, the likelihood of returning to community life, and economic status [34]. Patients often experience homesickness after 1 month of hospitalization and, thus, are relatively more likely to request to be discharged [23]. In patients with an extended PAC-CVD LOS (0–9 weeks), the NHI cost was the highest during weeks 4–6; it was significantly higher than that during weeks 0–3 and 7–9. The patients with stroke were discharged when the extended hospital stay was <9 weeks, resulting in significantly lower NHI costs than those during weeks 4–6. Although the patient factors affect whether a patient should be discharged, PAC-CVD LOS extensions are approved only by the NHIA. This result suggested that the submission of LOS extension applications is partly responsible for NHI cost reduction.

In our further analysis, we found that age and sex had a significant explanatory power for the NHI cost in the first 3 weeks: the higher the age, the lower the NHI cost in the first 3 weeks of hospitalization. This finding was similar to that of previous studies—compared with younger patients, older patients had more unstable medical conditions in the early stages of stroke, along with a higher rate of complication development and a higher likelihood to suspend rehabilitative interventions [8,35]. Here, the PAC-CVD program delivered high-intensity rehabilitation (three to five sessions per day), and the older patients had a lower tolerance for exercise, which might increase the early discharge risk. In addition, sex had a significant explanatory power for the NHI cost in the first 3 weeks of hospitalization. The NHI cost was higher for male patients than for female patients, indicating that females had relatively fewer days in the first 3 weeks of hospitalization. A study found that female patients had a stroke at an older age compared with male patients and that comorbidities during stroke were more common in female patients than in male patients, which affected the prognosis of rehabilitation negatively [36]. As mentioned above, age is a factor affecting the LOS, making female patients less willing to stay hospitalized. In terms of humanities and society, studies have shown that most family caregivers are women (including wives and daughters). In particular, in Chinese culture, female family members are often expected to take care of the health needs of other family members and perform household chores at home [37]. Moreover, in the Chinese culture, fulfilling responsibilities and obligations is an important oath to each other in marriage, and female spouses are more able to accept and practice them [38]. As such, in Taiwan, when male patients experience a stroke event, their female relatives often provide complete medical care needs. Therefore, male patients with stroke receive sufficient rehabilitative support and accept longer-term inpatient rehabilitation intervention. Notably, the current results demonstrated that functional performance measures (mRS, BI, 5MWS, and 6MWD) did not have significant explanatory power for the NHI cost. This result indicated that the PAC-CVD program under the per diem reimbursement system continuously improved the functional performance and walking ability of the included patients such that a lower NHI cost is incurred during physical function recovery. Therefore, the PAC-CVD program can aid patients in effectively regaining a functional outcome by incurring no additional healthcare costs and thus reducing the economic burden.

This study has some limitations. First, our sample was obtained from a single center. Nevertheless, this is one of the institutions that had the highest number of PAC cases in Southern Taiwan. A relevant large-scale, multicenter population-based study may confirm the current findings. Second, this was a retrospective study that reviewed medical records, observed phenomena, and made clinical recommendations. Future studies may further explore psychological, social, and humanistic factors using qualitative methods to understand the factors that affect postrehabilitation care further. Third, this study analyzed the effects of motor recovery on the NHI cost, illustrating the cost-effectiveness of the PAC-CVD program. However, in terms of clinical practice, the analysis of the allocation of medical resources, such as the number of hospital days under the limited availability of beds, is warranted. Despite these limitations, the current study may be considered a crucial contribution to the literature on functional outcomes, NHI costs, and the cost-effectiveness of PAC-CVD programs.

## 5. Conclusions

This study highlighted the cost-effectiveness of the PAC-CVD program for patients with stroke. Patients who received high-intensity rehabilitation continued to demonstrate improvements in functional outcomes from rehabilitation weeks 0 to 12. The PAC-CVD program provides per diem reimbursement and allows applications for program extension to control the NHI costs and thereby reduce the financial burden of medical care. Although age and sex demonstrated a significant impact on the NHI costs at the beginning of the PAC-CVD program, functional performance demonstrated no significant effects on the NHI costs at each hospitalization time point.

Thus, participating in the PAC-CVD program does not incur increased NHI costs but improves physical function. In other words, the PAC-CVD program is an effective, economical public health policy, which aids patients with stroke in regaining physical function.

## Figures and Tables

**Table 1:** Patient characteristics and prerehabilitation functional performance measures (*n* = 263).

Variables		Mean (*SD*) or *n* (%)
Demographics		
Age, years		63.74 (13.08)
Sex	Female	91 (34.6%)
	Male	172 (65.4%)
Clinical Attributes		
Stroke Type		
Ischemic		188 (71.5%)
Hemorrhagic		75 (28.5%)
Hypertension		247 (93.9%)
Coronary Artery Disease		76 (28.9%)
Diabetes Mellitus		112 (42.6%)
Hyperlipidemia		121 (46.0%)
Previous Stroke		53 (20.2%)
Common Risk Factors		
Smoking		59 (22.4%)
Drinking		29 (11.0%)
Prerehabilitation Functional Status		
mRS		3.66 (0.47)
BI		44.56 (22.23)
5MWS		0.1 (0.24)
6MWD		25.67 (67.64)
Medical Care of PAC-CVD Program		
PAC-CVD LOS, days		32.57 (15.28)
Weeks of Hospitalization		
0–3 Weeks		111 (42.2%)
4–6 Weeks		126 (47.9%)
7–9 Weeks		20 (7.6%)
10–12 Weeks		6 (2.3%)
Cost of NHI, USD		3189.95 (1501.07)

*SD*: standard deviation; mRS: modified Rankin scale; BI: Barthel index; 5MWS: 5-m walking speed; 6MWD: 6-min walking distance; PAC-CVD: postacute care cerebrovascular disease; LOS: length of stay; NHI: national health insurance.

**Table 2:** Functional performance measure scores and NHI cost over a PAC-CVD LOS of 0–3 weeks (*n* = 263).

	0–3 Weeks Functional Status		
Variables	W0	W3	*p*
mRS	3.66 (0.47)	3.02 (0.90)	<0.001 ***
BI	44.56 (22.23)	68.92 (24.40)	<0.001 ***
5MWS	0.1 (0.24)	0.29 (0.45)	<0.001 ***
6MWD	25.67 (67.64)	85.15 (126.87)	<0.001 ***
	Cost of NHI		
	Admitted	0–3 weeks	*p*
Cost of NHI, USD	-	1895.31 (251.47)	-

W0: prerehabilitation; W3: week 3 of PAC-CVD program; mRS: modified Rankin scale; BI: Barthel index; 5MWS: 5-m walking speed; 6MWD: 6-min walking distance; NHI: national health insurance. Values are expressed as mean (*SD*). *** Statistically significant (*p* < 0.001).

**Table 3:** Functional performance measure scores and NHI cost over a PAC-CVD LOS of 0–6 weeks (*n* = 152).

0–6 Weeks Functional Status						
Variables	W0	W3	W6	*F*	*p*	Pairwise Comparison
mRS	3.87 (0.33)	3.51 (0.61)	3.12 (0.73)	131.88 ***	<0.001	W0 > W3 ^a^, W0 > W6 ^a^, W3 > W6 ^a^
BI	34.38 (18.44)	56.58 (21.73)	67.89 (20.62)	327.51 ***	<0.001	W6 > W3 ^a^, W6 > W0 ^a^, W3 > W0 ^a^
5MWS	0.02 (0.10)	0.14 (0.42)	0.23 (0.34)	28.38 ***	<0.001	W6 > W3 ^a^, W6 > W0 ^a^, W3 > W0 ^a^
6MWD	6.59 (40.77)	32.97 (81.13)	65.54 (101.28)	54.60 ***	<0.001	W6 > W3 ^a^, W6 > W0 ^a^, W3 > W0 ^a^
Cost of NHI						
	Admitted	W3	W6	*F*	*p*	Pairwise Comparison
Cost of NHI, USD	-	2014.74 (84.32)	1843.27 (291.97)	6344.74 ***	<0.001	W3 > W6 ^a^, W6 > W0 ^a^, W3 > W0 ^a^

W0: prerehabilitation; W3: week 3 of PAC-CVD program; W6: week 6 of PAC-CVD program; mRS: modified Rankin scale; BI: Barthel index; 5MWS: 5-m walking speed; 6MWD: 6-min walking distance; W3 in cost of NHI: NHI cost during 0–3 weeks; W6 in cost of NHI: NHI cost during 4–6 weeks; NHI: national health insurance. Values are expressed as mean (*SD*). *** Statistically significant (*p* < 0.001). ^a^ Pairwise comparison significant.

**Table 4:** Functional performance measure scores and NHI cost over a PAC-CVD LOS of 0–9 weeks (*n* = 26).

0–9 Weeks Functional Status							
Variables	W0	W3	W6	W9	*F*	*p*	Pairwise Comparison
mRS	3.88 (0.33)	3.73 (0.45)	3.54 (0.51)	2.96 (0.77)	21.16 ***	<0.001	W0 > W3, W0 > W6 ^a^, W0 > W9 ^a^, W3 > W6, W3 > W9 ^a^, W6 > W9 ^a^
BI	33.27 (16.55)	51.35 (20.86)	58.46 (19.17)	74.62 (16.12)	72.31 ***	<0.001	W9 > W6 ^a^, W9 > W3 ^a^, W9 > W0 ^a^, W6 > W3 ^a^, W6 > W0 ^a^, W3 > W0 ^a^
5MWS	<0.001 (<0.001)	0.04 (0.14)	0.07 (0.22)	0.20 (0.28)	9.86 ***	0.001	W9 > W6 ^a^, W9 > W3 ^a^, W9 > W0 ^a^, W6 > W3, W6 > W0, W3 > W0
6MWD	<0.001 (<0.001)	9.35 (37.59)	21.98 (58.07)	61.31 (104.13)	7.66 **	0.004	W9 > W6, W9 > W3 ^a^, W9 > W0 ^a^, W6 > W3, W6 > W0, W3 > W0
Cost of NHI							
	Admitted	W3	W6	W9	*F*	*p*	Pairwise Comparison
Cost of NHI, USD	-	1993.31 (92.54)	2060.84 (44.57)	1928.40 (178.61)	2487.77 ***	<0.001	W6 > W9 ^a^, W3 > W9, W9 > W0 ^a^, W6 > W3 ^a^, W6 > W0 ^a^, W3 > W0 ^a^

W0: prerehabilitation; W3: week 3 of PAC-CVD program; W6: week 6 of PAC-CVD program; W9: week 9 of PAC-CVD program; mRS: modified Rankin scale; BI: Barthel index; 5MWS: 5-m walking speed; 6MWD: 6-min walking distance; W3 in cost of NHI: NHI cost during 0–3 weeks; W6 in cost of NHI: NHI cost during 4–6 weeks; W9 in cost of NHI: NHI cost during 7–9 weeks; NHI: national health insurance. Values are expressed as mean (*SD*). ** Statistically significant (*p* < 0.01). *** Statistically significant (*p* < 0.001). ^a^ Pairwise comparison significant.

**Table 5:** Functional performance measure scores and NHI cost over a PAC-CVD LOS of 10–12 weeks (*n* = 6).

	9–12 Weeks Functional Status				
Variables	W9		W12		
	Median (IQR)	Mean (*SD*)	Median (IQR)	Mean (*SD*)	*p*
mRS	3 (0.25)	3.17 (0.41)	3 (1)	2.67 (0.52)	0.08
BI	62.5 (13.75)	63.33 (8.76)	75 (16.25)	77.50 (12.15)	0.04 *
5MWS	0.05 (0.14)	0.07 (0.08)	0.17 (0.23)	0.14 (0.12)	0.07
6MWD	0 (38.75)	14.17 (22.45)	59 (58.25)	44.83 (30.92)	0.04 *
	Cost of NHI during 10 to 12 weeks				
Cost of NHI, USD	1695.53 (264.65)				

W9: week 9 of PAC-CVD program; W12: week 12 of PAC-CVD program; mRS: modified Rankin scale; BI: Barthel index; 5MWS: 5-m walking speed; 6MWD: 6-min walking distance; NHI: national health insurance. Values are expressed as the median and interquartile range (IQR), i.e., 3 (0.25) means the median is 3 and the difference between the third quartile and the first quartile is 0.25. The value of the cost of national health insurance is expressed as mean (*SD*). * Statistically significant (*p* < 0.05).

**Table 6:** Hierarchical multiple regression for the impact of week 3 functional performance on the NHI cost (*n* = 263).

Model Summary							
Model	*R*	*R* ^2^	Adjusted *R*^2^	*R*^2^ Change	*F* Change	Sig. *F* Change	Durbin-Watson
1	0.208	0.043	0.036	0.043	5.858	0.003	-
2	0.263	0.069	0.032	0.026	0.879	0.535	-
3	0.407	0.166	0.119	0.097	7.175	<0.001	1.552
Predictive Factor Input							
Model	Predictor			*B*	*β*	*t*	*p*
1	Constant			59,524.25	-	24.14	<0.001
	Age			−75.79	−0.133	−2.162	0.032 *
	Sex			2153.20	0.138	2.238	0.026 *
2	Constant			56,156.05	-	15.95	<0.001
	Age			−74.55	−0.131	−1.874	0.062
	Sex			2576.49	0.165	2.487	0.014 *
	Stroke Type			31.11	0.002	0.025	0.980
	Previous Stroke			1675.62	0.090	1.440	0.151
	Hypertension			2474.72	0.080	1.271	0.205
	Coronary Artery Disease			534.77	0.033	0.515	0.607
	Diabetes Mellitus			−770.90	−0.051	−0.806	0.421
	Hyperlipidemia			−687.58	−0.046	−0.669	0.504
	Smoking			334.69	0.019	0.257	0.797
	Drinking			−2207.76	−0.093	−1.358	0.176
3	Constant			56,588.12	-	9.576	<0.001
	Age			−110.62	−0.194	−2.836	0.005 **
	Sex			2076.54	0.133	2.059	0.041 *
	Stroke Type			−554.81	−0.034	−0.468	0.640
	Previous Stroke			1849.77	0.100	1.660	0.098
	Hypertension			2391.33	0.077	1.268	0.206
	Coronary Artery Disease			496.01	0.030	0.494	0.622
	Diabetes Mellitus			−531.20	−0.035	−0.579	0.563
	Hyperlipidemia			−60.69	−0.004	−0.061	0.951
	Smoking			637.66	0.036	0.510	0.611
	Drinking			−2144.91	−0.090	−1.379	0.169
	mRS			1571.62	0.189	1.764	0.079
	BI			−40.13	−0.132	−1.352	0.178
	5MWS			124.40	0.008	0.073	0.942
	6MWD			−2.06	−0.035	−0.328	0.743

Adjusted *R*^2^*:* adjusted goodness-of-fit measure for the regression model; *B*: unstandardized coefficient; *β*: standardized coefficient; mRS: modified Rankin scale; BI: Barthel index; 5MWS: 5-m walking speed; 6MWD: 6-min walking distance. Model 1 includes patient demographics (age and sex). Model 2 is the second after model 1 and includes demographics (age and sex), clinical attributes (stroke type, stroke history, hypertension, coronary artery disease, diabetes mellitus, and hyperlipidemia), and common risk factors (smoking and drinking). Model 3 is the third after model 2 and includes demographics (age and sex), clinical attributes (stroke type, stroke history, hypertension, coronary artery disease, diabetes mellitus, and hyperlipidemia), common risk factors (smoking and drinking), and functional performance (mRS, BI, 5MWS, and 6MWD). Dependent variable: NHI cost at week 3. * Statistically significant (*p* < 0.05). ** Statistically significant (*p* < 0.01).

## Data Availability

The data presented in this study are available on request from the corresponding author.

## References

[1] Feigin V.L., Forouzanfar M.H., Krishnamurthi R., Mensah G.A., Connor M., Bennett D.A., Moran A.E., Sacco R.L., Anderson L., Truelsen T. (2014). Global and Regional Burden of Stroke During 1990-2010: Findings from the Global Burden of Disease Study 2010. Lancet.

[2] Patel A., Berdunov V., King D., Quayyum Z., Wittenberg R., Knapp M. (2017). Executive Summary Part 2: Burden of Stroke in the Next 20 Years and Potential Returns from Increased Spending on Research.

[3] Hsieh C.-Y., Lee T.-H., Chang K.-C., Taiwan Stroke Society (2014). A Nationwide Plan for Postacute Care of Stroke in Taiwan. Int. J. Stroke.

[4] Chien S.H., Sung P.Y., Liao W.L., Tsai S.W. (2020). A Functional Recovery Profile for Patients with Stroke Following Post-Acute Rehabilitation Care in Taiwan. J. Formos. Med. Assoc..

[5] Hsieh C.Y., Tsao W.C., Lin R.T., Chao A.C. (2018). Three Years of the Nationwide Post-Acute Stroke Care Program in Taiwan. J. Chin. Med. Assoc..

[6] Lai C.L., Tsai M.M., Luo J.Y., Liao W.C., Hsu P.S., Chen H.Y. (2017). Post-Acute Care for Stroke—A Retrospective Cohort Study in Taiwan. Patient Prefer. Adherence.

[7] Peng L.-N., Lu W.-H., Liang C.-K., Chou M.-Y., Chung C.-P., Tsai S.-L., Chen Z.-J., Hsiao F.-Y., Chen L.-K., Lin C.-S. (2017). Functional Outcomes, Subsequent Healthcare Utilization, and Mortality of Stroke Postacute Care Patients in Taiwan: A Nationwide Propensity Score-Matched Study. J. Am. Med. Dir. Assoc..

[8] Chou H.Y., Lo Y.C., Tsai Y.W., Shih C.L., Yeh C.T. (2021). Increased Anxiety and Depression Symptoms in Post-Acute Care Patients with Stroke during the COVID-19 Pandemic. Int. J. Environ. Res. Public Health.

[9] Chu C.L., Lee T.H., Chen Y.P., Ro L.S., Hsu J.L., Chu Y.C., Chen C.K., Pei Y.C. (2022). Recovery of Walking Ability in Stroke Patients through Postacute Care Rehabilitation. Biomed J..

[10] Preston E., Ada L., Dean C., Stanton R., Waddington G. (2011). What is the Probability of Patients who are Nonambulatory after Stroke Regaining Independent Walking? a Systematic Review. Int. J. Stroke.

[11] Rakesh N.K., Boiarsky D., Athar A., Hinds S., Stein J. (2019). Post-Stroke Rehabilitation: Factors Predicting Discharge to Acute Versus Subacute Rehabilitation Facilities. Medicine.

[12] Chiu C.C., Wang J.J., Hung C.M., Lin H.F., Hsien H.H., Hung K.W., Chiu H.C., Yeh S.C., Shi H.Y. (2021). Impact of Multidisciplinary Stroke Post-Acute Care on Cost and Functional Status: A Prospective Study Based on Propensity Score Matching. Brain Sci..

[13] Chen Y.C., Yeh Y.J., Wang C.Y., Lin H.F., Lin C.H., Hsien H.H., Hung K.W., Wang J.D., Shi H.Y. (2022). Cost Utility Analysis of Multidisciplinary Postacute Care for Stroke: A Prospective Six-Hospital Cohort Study. Front. Cardiovasc. Med..

[14] Banks J.L., Marotta C.A. (2007). Outcomes Validity and Reliability of the Modified Rankin Scale: Implications for Stroke Clinical Trials: A Literature Review and Synthesis. Stroke.

[15] Shah S., Vanclay F., Cooper B. (1989). Improving the sensitivity of the Barthel Index for stroke rehabilitation. J. Clin. Epidemiol..

[16] Mahoney F.I., Barthel D.W. (1965). Functional evaluation: The Barthel Index. Md. State Med. J..

[17] Salbach N.M., Mayo N.E., Higgins J., Ahmed S., Finch L.E., Richards C.L. (2001). Responsiveness and predictability of gait speed and other disability measures in acute stroke. Arch. Phys. Med. Rehabil..

[18] Fulk G.D., He Y., Boyne P., Dunning K. (2017). Predicting Home and Community Walking Activity Poststroke. Stroke.

[19] Kline R.B. (2005). Principles and Practice of Structural Equation Modeling.

[20] Hair J.F., Sarstedt M., Hopkins L., Kuppelwieser V.G. (2014). Partial Least Squares Structural Equation Modeling (Pls-Sem): An Emerging Tool in Business Research. Eur. Bus. Rev..

[21] Tung Y.J., Huang C.T., Lin W.C., Cheng H.H., Chow J.C., Ho C.H., Chou W. (2021). Longer length of post-acute care stay causes greater functional improvements in poststroke patients. Medicine.

[22] Wang C.Y., Chen Y.R., Hong J.P., Chan C.C., Chang L.C., Shi H.Y. (2017). Rehabilitative post-acute care for stroke patients delivered by per-diem payment system in different hospitalization paths: A Taiwan pilot study. Int. J. Qual. Health Care.

[23] Wang C.Y., Miyoshi S., Chen C.H., Lee K.C., Chang L.C., Chung J.H., Shi H.Y. (2020). Walking ability and functional status after post-acute care for stroke rehabilitation in different age groups: A prospective study based on propensity score matching. Aging.

[24] Quinn T., Harrison J.K., Arthur M. (2013). Assessment scales in stroke: Clinimetric and clinical considerations. Clin. Interv. Aging.

[25] Wolfe C.D., Taub N., Woodrow E., Burney P.G. (1991). Assessment of Scales of Disability and Handicap for Stroke Patients. Stroke.

[26] Liu F., Tsang R.C., Zhou J., Zhou M., Zha F., Long J., Wang Y. (2020). Relationship of Barthel Index and its Short Form with the Modified Rankin Scale in acute stroke patients. J. Stroke Cerebrovasc. Dis..

[27] Schmid A., Duncan P., Studenski S., Lai S., Richards L., Perera S., Wu S.S. (2007). Improvements in Speed-Based Gait Classifications Are Meaningful. Stroke.

[28] Brincks J., Nielsen J.F. (2012). Increased power generation in impaired lower extremities correlated with changes in walking speeds in sub-acute stroke patients. Clin. Biomech..

[29] In T.S., Jung J.H., Jung K.S., Cho H.Y. (2021). Effect of Sit-to-Stand Training Combined with Taping on Spasticity, Strength, Gait Speed and Quality of Life in Patients with Stroke: A Randomized Controlled Trial. Life.

[30] Vistamehr A., Kautz S.A., Bowden M.G., Neptune R.R. (2019). The influence of locomotor training on dynamic balance during steady-state walking post-stroke. J. Biomech..

[31] Newell A.M., VanSwearingen J.M., Hile E., Brach J.S. (2012). The Modified Gait Efficacy Scale: Establishing the Psychometric Properties in Older Adults. Phys. Ther..

[32] Makizako H., Shimada H., Yoshida D., Anan Y., Ito T., Doi T., Tsutsumimoto K., Uemura K., Brach J.S., Suzuki T. (2014). Reliability and Validity of the Japanese Version of the Modified Gait Efficacy Scale. J. Jpn. Phys. Ther. Assoc..

[33] Chang K.V., Chen K., Chen Y., Lien W., Chang W., Lai C., Wu C., Chen C., Chen Y., Wu W. (2022). A Multicenter Study to Compare the Effectiveness of the Inpatient Post Acute Care Program Versus Traditional Rehabilitation for Stroke Survivors. Sci. Rep..

[34] Longley V., Peters S., Swarbrick C., Bowen A. (2019). What factors affect clinical decision-making about access to stroke rehabilitation? A systematic review. Clin. Rehabil..

[35] Hung C.Y., Wu W.T., Chang K.V., Wang T.G., Han D.S. (2017). Predicting the length of hospital stay of post-acute care patients in Taiwan using the Chinese version of the continuity assessment record and evaluation item set. PLoS ONE.

[36] Koton S., Telman G., Kimiagar I., Tanne D. (2013). Gender differences in characteristics, management and outcome at discharge and three months after stroke in a national acute stroke registry. Int. J. Cardiol..

[37] Mak A.K., Mackenzie A., Lui M.H. (2007). Changing needs of Chinese family caregivers of stroke survivors. J. Clin. Nurs..

[38] Qiu X., Sit J.W., Koo F.K., Qiu O.X. (2017). The influence of Chinese culture on family caregivers of stroke survivors: A qualitative study. J. Clin. Nurs..

